# Model citizen - Authors’ reply

**DOI:** 10.1016/S2214-109X(17)30338-8

**Published:** 2017-10-01

**Authors:** Oliver J Brady, Hannah C Slater, Peter Pemberton-Ross, Edward Wenger, Richard J Maude, Azra C Ghani, Melissa A Penny, Jaline Gerardin, Lisa J White, Nakul Chitnis, Ricardo Aguas, Simon I Hay, David L Smith, Erin M Stuckey, Emelda A Okiro, Thomas A Smith, Lucy C Okell

**Affiliations:** Centre for the Mathematical Modelling of Infectious Diseases Department of Infectious Disease Epidemiology, and Malaria Modelling Consortium, London School of Hygiene & Tropical Medicine, London, UK; MRC Centre for Outbreak Analysis, and Modelling, Department of Infectious Disease Epidemiology, Imperial College, London W2 1PG, UK; Swiss Tropical and Public Health Institute, Basel, Switzerland; University of Basel, Basel, Switzerland; Institute for Disease Modeling, Bellevue, WA, USA; Mahidol Oxford Tropical Medicine Research Unit, Faculty of Tropical Medicine, Mahidol University, Bangkok, Thailand; Centre for Tropical Medicine and Global Health, University of Oxford, Oxford, UK; Harvard TH Chan School of Public Health, Harvard University, Boston, MA, USA; MRC Centre for Outbreak Analysis and Modelling, Department of Infectious Disease Epidemiology, Imperial College, London W2 1PG, UK; Swiss Tropical and Public Health Institute, Basel, Switzerland; University of Basel, Basel, Switzerland; Institute for Disease Modeling, Bellevue, WA, USA; Mahidol Oxford Tropical Medicine Research Unit, Faculty of Tropical Medicine, Mahidol University, Bangkok, Thailand; Centre for Tropical Medicine and Global Health, University of Oxford, Oxford, UK; Swiss Tropical and Public Health Institute, Basel, Switzerland; University of Basel, Basel, Switzerland; Mahidol Oxford Tropical Medicine Research Unit, Faculty of Tropical Medicine, Mahidol University, Bangkok, Thailand; Centre for Tropical Medicine and Global Health, University of Oxford, Oxford, UK; Oxford Big Data Institute, Li Ka Shing Centre for Health Information and Discovery, University of Oxford, Oxford, UK; Malaria Modelling Consortium, University of Washington, Seattle, WA, USA; Institute for Health Metrics and Evaluation, University of Washington, Seattle, WA, USA; Institute for Health Metrics and Evaluation, University of Washington, Seattle, WA, USA; Malaria Modelling Consortium, Bill & Melinda Gates Foundation, Seattle, WA, USA; Malaria Modelling Consortium, Bill & Melinda Gates Foundation, Seattle, WA, USA; Kemri Wellcome Trust Research Programme, Nairobi, Kenya; Swiss Tropical and Public Health Institute, Basel, Switzerland; University of Basel, Basel, Switzerland; MRC Centre for Outbreak Analysis and Modelling, Department of Infectious Disease Epidemiology, Imperial College, London W2 1PG, UK

Tom Peto and colleagues point out, reasonably, that the effect of mass drug administration (MDA) on malaria could be affected substantially by patterns of human movement, and that our Article^1^ does not consider the effects of the specific patterns of movement they observed in Cambodia and west Africa. The purpose of our Article^1^ was to derive general results about the possible effect of MDA and to test how robust these are to the assumptions in different models, so we avoided assumptions about population movement that are specific to any particular place. Migration is among several factors that the different groups in our collaboration have investigated as potential modifiers of the effects of mass treatment in specific situations. For those analyses, we have used field data or realistic assumptions of migration rates.^2–4^ Human migration is a particularly complicated modifier; the endemicity of malaria in the places from which immigrants or temporary visitors originate could be important, not just the season and extent of migration.

A thorough empirical investigation of the implications of different patterns of migration for the effect of MDA would require a prohibitively complex set of field trials. Corresponding *in silico* analyses, parameterised with local field data, are more feasible but still represent an extensive piece of research in their own right, which is well beyond the scope of our Article.^1^ Some of our research groups use extensive field data collection for this exact purpose.^5^ The questions addressed in our Article^1^ were raised by WHO for their Evidence Review Group on mass treatment of malaria, which contains many experts who work directly on MDA interventions. Furthermore, several ongoing modelling exercises are being carried out by the authors of our Article^1^ in collaboration with control programmes to assess the potential effect of MDA in specific settings.^6^ We agree with Peto and colleagues on the importance of modelling groups and field researchers working closely together to better inform models and improve predictions of intervention outcomes. Involvement of modellers in trial design and operational planning for specific interventions allows questions to be framed as accurately as possible for relevant geographies and broader policy recommendations, and we hope that the number of these exercises will increase in the coming years.
